# Higher-Order Conditioning in the Spatial Domain

**DOI:** 10.3389/fnbeh.2021.766767

**Published:** 2021-11-23

**Authors:** Youcef Bouchekioua, Yutaka Kosaki, Shigeru Watanabe, Aaron P. Blaisdell

**Affiliations:** ^1^Department of Neuropharmacology, Graduate School of Medicine, Hokkaido University, Sapporo, Japan; ^2^Department of Psychology, Waseda University, Tokyo, Japan; ^3^Department of Psychology, Keio University, Tokyo, Japan; ^4^Department of Psychology and Brain Research Institute, University of California, Los Angeles, Los Angeles, CA, United States

**Keywords:** higher-order conditioning, cognitive map, spatial memory, associative learning, inference, spatial integration, navigation

## Abstract

Spatial learning and memory, the processes through which a wide range of living organisms encode, compute, and retrieve information from their environment to perform goal-directed navigation, has been systematically investigated since the early twentieth century to unravel behavioral and neural mechanisms of learning and memory. Early theories about learning to navigate space considered that animals learn through trial and error and develop responses to stimuli that guide them to a goal place. According to a trial-and error learning view, organisms can learn a sequence of motor actions that lead to a goal place, a strategy referred to as response learning, which contrasts with place learning where animals learn locations with respect to an allocentric framework. Place learning has been proposed to produce a mental representation of the environment and the cartesian relations between stimuli within it—which Tolman coined the cognitive map. We propose to revisit some of the best empirical evidence of spatial inference in animals, and then discuss recent attempts to account for spatial inferences within an associative framework as opposed to the traditional cognitive map framework. We will first show how higher-order conditioning can successfully account for inferential goal-directed navigation in a variety of situations and then how vectors derived from path integration can be integrated via higher-order conditioning, resulting in the generation of higher-order vectors that explain novel route taking. Finally, implications to cognitive map theories will be discussed.

## Introduction

O’Keefe and Nadel (1978) elaborated on the separate systems that utilize bottom up vs. top-down processes for navigation, each having a separate neural basis. The taxon system is bottom up and utilizes processes of path integration and associative learning (e.g., beacon homing). The locale system is top down and involves the allocentric representation of space as the cognitive map. One important tenet of the cognitive map theory is that its manifestation should not be explained by path integration (O’Keefe and Nadel, 1978; Bennett, 1996; Singer et al., 2006), the process by which animals go back to a home nest using a direct vector after a random journey, first reported by Darwin (1873) as dead-reckoning. This basic and automatic ability to compute a direct home vector can be solely based on internal information such as vestibular and proprioceptive perception (Collett et al., 1998; Müller and Wehner, 1988; Schatz et al., 1999; Etienne and Jeffery, 2004). Constantly updating distance and direction from the current position to the start place during a journey enables direct return to the start place simply by following the last computed vector, even if this goal-vector points toward a path never experienced. This can be achieved through vector arithmetic where a journey is decomposed into vectors, with the first vector taking origin at the start place, and with any change of direction triggering the calculation of a new vector. Path integration, along with other egocentric based navigation strategies, is part of the taxon system, as opposed to the map-based locale system responsible for allocentric navigation strategies (O’Keefe and Nadel, 1978).

Contrary to the traditional view of cognitive map theory that a detailed spatial map is necessary for spatial inferences, higher-order conditioning is a bottom-up associative process that provides an alternative means to learn relationships between events without direct experience, and that enables spatial inferences without a detailed spatial representation connecting all spatial locations visited during prior navigation. Higher-order conditioning was first discovered by Pavlov (1927) and consists of the conditioning that can occur to a cue (e.g., a conditional stimulus or CS) even when that CS had not been directly paired with a rewarding outcome (e.g., an unconditional stimulus or US). For instance, in a sensory preconditioning (SPC) procedure, first discovered by Pavlov (Kimmel, 1977) and later confirmed by Brogden (1939), a CS (CS2) is first paired with another CS (CS1) in stage 1, followed by a second stage during which CS1 is paired with an unconditional stimulus (US). When testing CS2 in stage 3, a conditional response is observed even though CS2 had not been directly paired with the US. Higher-order conditioning has been observed in a broad range of species, including, but not limited to, crickets (Matsumoto et al., 2013), molluscs (Kojima et al., 1998), drosophila (Heisenberg et al., 2001), honeybees (Muller et al., 2000), pigeons (Sawa et al., 2005), rodents (Brogden, 1939), and humans (Brogden, 1947), and suggests that it is a fundamental mechanism of learning in organisms possessing a central nervous system.

## Spatial Integration

The following is a representative overview of studies specifically designed to assess higher-order conditioning in the spatial domain, in different species, namely, pigeons, rats and humans, and with spatial information of various nature (intra-maze cues, extra-maze cues and boundaries).

### In Pigeons With Intra-Maze Cues

**FIGURE 1:**
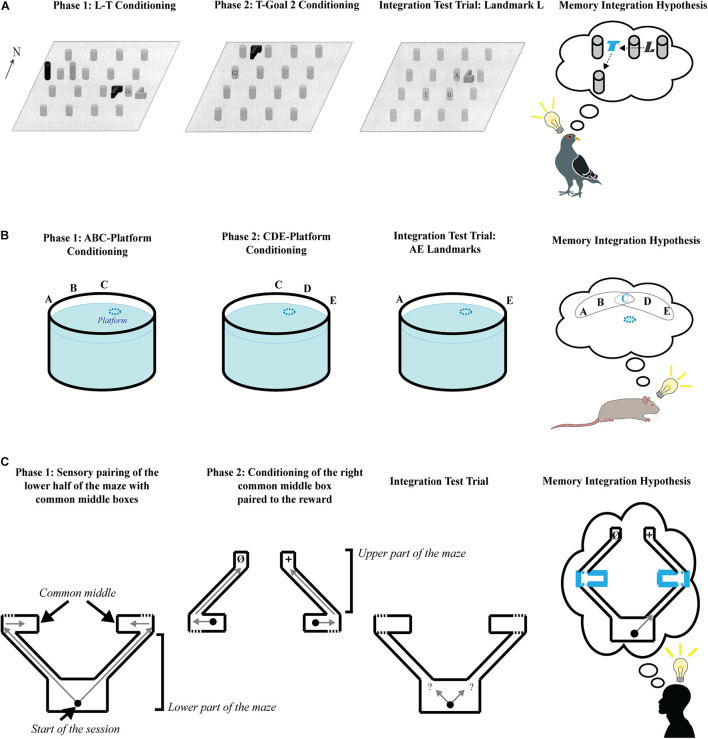
Schematic representation of spatial integration tasks following the sensory preconditioning procedure. Each experiment consists of two learning phases followed by a test phase. A schematic representation of the hypothetical result of memory integration is proposed for each study. **(A)** Diagram of the experimental design used by Blaisdell and Cook (2005), showing the configuration of the 4 × 4 grid of gravel-filled cups, the hidden food (G), and the landmarks (T, L, and two foils). The left panel show the spatial arrangement of the consistent landmarks (T and L), goal 1 (G1), and inconsistent landmarks (cylindrical foils) during Phase 1. The second panel (from the left) shows the spatial arrangement of landmark T to goal 2 (G2) during Phase 2. The third panel (from the left) shows the spatial arrangement of landmark L and the potential locations of search during the integration test. Letters on bottom panel: I = predicted cup for choices guided by the L → T goal 2 hierarchical map, A = predicted cup for choices guided by the phase 1 L→ goal 1 vector, and G = predicted cup for choices guided by a generalization to L of the T→ goal 2 vector. Reprinted with permission of the authors. The last panel (from the left) represents the hypothetical integration of separately formed memories, where the common element (i.e., landmark T) appears in cyan color. **(B)** Schematic representation of the pool and the configuration of the landmarks used by Chamizo et al. (2006) in each phase of the task. The blue dashed-line circle represents the hidden platform under opaque water. A, B, C, D, and E are extra-maze visual cues. The last panel (from the left) represents the hypothetical integration of separately learned memories, where the common element (i.e., landmark C) appears in cyan color. **(C)** Schematic representation of the 3D virtual task used by Bouchekioua et al. (2013) for each phase of the task. The black dot represents the start place in all panels, and the gray arrows of the two first panels (from the left) show the journeys experienced by the participants. In the second panel (from the left), Ø = no reward is present in the end box, + = a reward is present in the end box. The gray arrow question marks in the third panel (from the left) show the two possible choices during the test. The last panel (from the left) represents the hypothetical integration of separately formed memories, where the common elements (i.e., the common middle boxes) are in cyan bold lines, and the gray arrow represents the choice leading to the reward.

Assessment of the role of higher-order conditioning in goal-directed navigation was instigated by Blaisdell and Cook (2005) in a study that involved pigeons navigating in an open area with intra-maze cues as CSs (Figure 1A). During the first phase, pigeons were trained to find a hidden food reward (G1) located between two intra-maze cues, L and T, with their spatial relationship with respect to each other kept constant across trials. However, their position in the arena was changed stochastically between trials, thus neutralizing the use of spatial information provided by room cues to find G1. During the second phase, L was removed, and pigeons learned to find the hidden food reward (G2) in a new location with which T kept a constant spatial relationship. The animals were then tested in the presence of L alone, which had never directly been paired with G2. If we consider that pigeons learned the L→T vector in Phase 1, and the T→G2 vector during Phase 2, integrating these two vectors through the common element they share (i.e., T) should enable pigeons to infer vector L→G2. Consistent with a strategy based on the integration of these spatial relationships, pigeons spent more time searching for food at the location based on the inferred L→G2 vector than at other locations (with the exception of the location of G1 acquired during Phase 1). While the procedure used in this experiment does not strictly follow the sensory preconditioning procedure, where neutral stimuli are originally paired in the absence of a US, it recapitulates the critical feature of higher-order conditioning, being that separately learned associations can be integrated if they share a common element or event. Overcoming this limitation, Sawa et al. (2005) replicated this finding in a 2D version of the task using a touchscreen panel, where the US (i.e., food reward) was not present during the first phase. Spatial integration has been confirmed in humans, using a 3D virtual reality version of the task, where the reward was presented only during Phase 2 (Molet et al., 2011).

### In Rats With Extra-Maze Cues

In a similar study, the ability to integrate a set of extra-maze cues in a Morris-water maze task was assessed in rats (Chamizo et al., 2006) by training the animals to find a hidden platform using a set of three extra-maze cues (e.g., A, B and C) during a first phase (Figure 1B). In a second phase, a second set of cues including C (e.g., C, D E) was paired with the hidden platform at the same location. When tested with one cue from each Phase (e.g., A and E), without the presence of the common landmark C, rats searched more for the platform in the quadrant where its location would be inferred if the missing cues were retrieved by an integration of both set of cues separately learned. Moreover, the animals performed as well as rats trained with all cues present on all trials. Rats trained without a common cue C between the two sets of cues (e.g., A, B and C in Phase 1 and D, E and F in Phase 2) failed to search for the platform in the correct quadrant more than a chance amount of time.

### In Humans With Boundaries

Consistent with the studies presented above, it has been shown in humans that boundaries of an environment can be used as CSs and follow the rules of higher-order conditioning (Bouchekioua et al., 2013). In a 3D virtual maze, human participants were trained to explore two paths connecting a starting room with two separate adjoining boxes (“Common middle boxes,” see Figure 1C). During a second Phase, the participants directly started from one of the common middle boxes on half of the trials, and from the other common middle box on the second half of the trials. Each common middle box was connected to an end box via a pathway. The participants were allowed to navigate from a common middle box to an end box during Phase 2 and found a reward (a virtual treasure Chest) in only one of the end boxes, while the remaining end box was left empty. In a subsequent one-shot test, the participants were placed in the starting room and asked to find the treasure. Most participants chose the pathway that led to treasure, suggesting that they were able to infer that this pathway leads to the goal-place, even though they had never directly experienced the reward from this pathway. This suggests that they had integrated each of the separately learned sets of geometrical information that shared a common element. A control group directly started outside of the common middle boxes in Phase 2 and were not allowed to associate the common middle boxes to their respective end box. While they had equal experience of the end boxes, and found the treasure in only one of them, they performed at chance level at test.

Taken together, these studies suggest that higher-order conditioning enables the use of various types of spatial CS (intra-maze, extra-maze, boundaries) for flexible goal-directed navigation where the complete route leading to a goal place is explored in a piecemeal fashion. The mental integration of these spatial routes using higher order conditioning processes supports spatial inference, such as the selection of the shortest route or a correct route that leads to a reward and suggests that the taxon-system is sufficient for supporting flexible goal-directed navigation. The traditional view of the cognitive map is that it enables the linking “together conceptually parts of an environment which have never been experienced at the same time” (O’Keefe and Nadel, 1978; Poucet, 1993), and can be used to mentally retrieve parts of the environment that are outside of the field of perception (Pick and Rieser, 1982). Contrasting with this view, a recent study found that rats flexibly adapted to changes in connectivity between four familiar rooms, while CA1 hippocampal place-cells did not respond to changes in connectivity (Duvelle et al., 2021). Thus, associative learning and cognitive map theories provide satisfactory accounts for inferential goal-directed navigation supported by mental integration of separately explored, but familiar routes. The ability of taking a novel route never fully explored either within a single session, or in a piecemeal fashion during separate sessions, has been predicted by the cognitive map theory but not by associative learning theories. It is indeed difficult to conceive how a stimulus that had never been perceived could be associated with a goal-place. While the cognitive map theory predicted the ability of novel route taking, it did not provide any mechanism for it. How could places never explored be integrated into a mental representation of the environment? We present next a study that implies both spatial integration and novel route taking in rats within the same experiment (Roberts et al., 2007), and show how a combination of two strategies from the taxon-system, namely higher-order conditioning and path integration, can explain these results.

## Novel Route Taking

**FIGURE 2:**
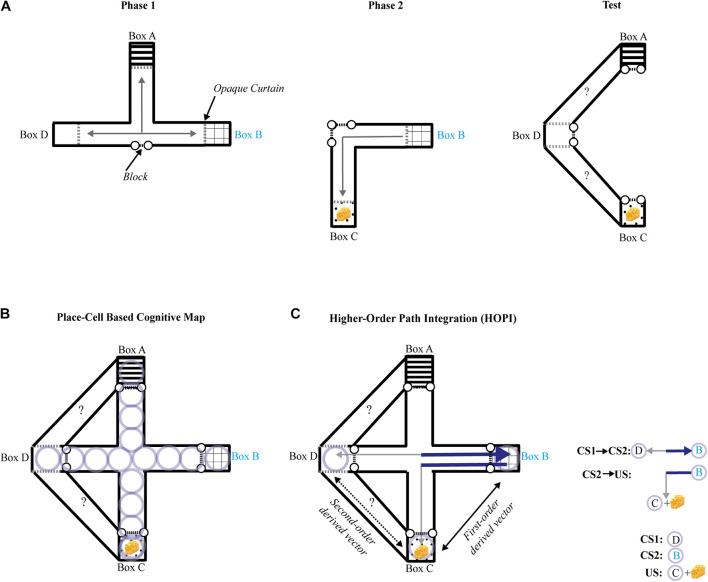
**(A)** Schematic representation of the maze and procedure used by Roberts et al. (2007) seen from above during each phase of the experiment. Black dashed lines with dots represent barriers, and gray arrows represent the paths traveled by rats during training phases 1 and 2 (left and middle panels). The right panel shows the configuration of the maze during the test phase, where two new adjacent paths were opened, while access to the rest of the maze was blocked. The question marks represent the possible choices during the test. Dashed gray lines represent opaque curtains, the common element (box B) is annotated in cyan characters. The yellow cheese represents the reward. **(B)** Schematic representation of the maze used by Roberts et al. (2007) seen from above during the test phase. Light purple circles represent place-cell representations formed during the training phases. **(C)** Schematic representation of the maze used by Roberts et al. (2007) seen from above during the test phase. The underlying strategy based on higher-order path integration (HOPI, see Bouchekioua et al., 2021) is represented as follows: solid gray arrows represent direct vectors formed during the training phases 1 and 2; solid black arrow represents a first-order derived vector computed by vector arithmetic between direct vectors. The dark blue section of the direct vectors in bold lines represents the common segment they share; dashed black arrow represents a second-order derived vector resulting from vector arithmetic between a direct vector and a first-order derived vector.

Several criteria have to be met when assessing novel route taking: (1) The novel shortcut should be performed in a one-shot trial; (2) It should not result from a behavior previously reinforced (e.g., response learning); (3) Extra-maze and intra-maze cues directly paired with a goal-place should not be available during the novel shortcut test (i.e., beacon homing must be prevented); and (4) Taking a novel route should not be explained by simple path integration. Roberts et al. (2007) tested the ability of rats to a take novel route in an experiment designed to meet all the above criteria. To that aim, the authors used an enclosed maze covered by a ceiling and the maze was rotated to a random orientation (north, south, east, or west) before each trial, thus neutralizing extra-maze cues, and no distinct intra-maze cue was available during the test session. To ensure that the animals could not associate any room cues with the goal-place while being moved from their cage to the maze, they were transported in an opaque box, and released directly inside the maze. White noise was played to cover any sound that could serve as a directional or positional cue, and the maze was washed with a vinegar and water solution after each trial to eliminate any olfactory trace that may have been left by the rat. The first phase of training took place in a restricted area of the entire maze, composed of only three boxes (A, B, and D; see Figure 2A) and their connecting alleys. Rats were first allowed to consume a small portion of food in one box before being placed in one of the two remaining boxes. Across the first phase of training, rats learned to directly find the food reward from the two remaining boxes, with all possible combinations of boxes serving as a starting place or goal-place. During the second phase of training, all rats consumed a small amount of food in the new box C, but only half of them, constituting the experimental group, were then placed in B and allowed to go back to C. Blocks were introduced to prevent rats from exploring any of the maze beyond the internal alleys that directly connected B to C. During the test, all rats were pre-fed in goal place C and transported to D. The access to internal alleys of the maze were blocked, and the animals were given a choice between two new adjacent paths never previously explored or perceived, only one of them leading to goal place C. Even though animals had no chance to directly connect D to C during previous training, only those of the experimental group successfully chose the correct path leading from D to C by making a right turn. It is worth noting that reinforcement of a left turn in Phase 2 neutralizes a simple egocentric account, as the correct turn during the test trial was to the right side. As predicted by cognitive map theory, the results provide unequivocal evidence that rats were able to take a novel route in goal-directed navigation, without using simple path integration, response learning, extra-, or intra-maze cues, and cannot be explained by trial-and-error learning. It is unclear how referring to a spatial representation of the maze could help in solving the novel route task. Place cells, neurons in the hippocampus that have the property of manifesting a maximal firing rate for a specific area of a familiar environment, have been proposed to support prospective planning of spatial navigation. Serial activations of place cells coding for adjacent places and covering a familiar environment has thus been proposed as a mechanism of cognitive mapping for flexible goal-directed navigation (O’Keefe and Nadel, 1978; Pfeiffer and Foster, 2013). Rats had, however, no occasion to form a map-like representation of the correct route in the experiment of Roberts et al. (2007) using place cells, as they explored none of the two new adjacent paths available during the test (Figure 2B)^1^. Recent studies suggest that place-cells are not involved in route planning, but rather play a role in discriminating alternative routes (Grieves et al., 2016) irrespective of their relationship with the reward (Duvelle et al., 2019). We recently proposed a model called higher-order path integration (HOPI) to explain spatial inferences such as that shown by the rats in Roberts et al. (Bouchekioua et al., 2021). HOPI combines two strategies that O’Keefe and Nadel (1978) assign to the taxon system, path integration and higher-order conditioning, and presumes that place-cell activity reflects known routes rather than inferred, unfamiliar ones. HOPI can explain novel shortcut behavior if we consider the possibility that vectors derived from path integration can be stored in reference memory as direct vectors. Separate direct vectors can be integrated into first-order derived vectors if they share a common segment or connecting point. Furthermore, separate first-order derived vectors that share a common segment or connecting point can be further integrated into second-order derived vectors through vector arithmetic (Etienne et al., 1998). Such integrated first-order and second-order derived vectors could then be used to navigate along novel routes to reach goals (Figure 2C). HOPI explains how vectors, whether direct or derived from path integration, can be integrated the same way other types of cues are, that is, following rules higher-order conditioning processes. Specifically, associations sharing a common element can be mentally connected. In addition, HOPI applies vector arithmetic (i.e., addition and/or subtraction) to vectors sharing a common element, which results in the generation of higher-order vectors that connect places with approximated distance and directional information, even if the places covering these mentally computed vectors have never been explored. In other words, HOPI informs the shortest direction and distance between two places, without requiring prior exploration of this novel shortcut. Importantly, unlike the explicit map-like mental representation envisioned by Tolman (1948) with neural underpinnings proposed by O’Keefe and Nadel (1978), spatial relationships of the environment are built up from bottom-up associative and path integration processes (i.e., the taxon system) to form an implicit knowledge of cartesian space and the objects, events, and places within it that can be accessed at any time to derive navigation choices, even for unfamiliar routes. To clarify this subtle difference imagine the following scenario. A person is placed in a familiar location with which they have had a lot of prior experience, such as a city street in their home town. Let’s imagine the person was asked where a building is located in comparison to their current position, and that they had never previously traveled from their current location to the target building, thus, the route connecting them is unfamiliar. According to cognitive map theory, the person could draw with pen and paper a top down map of the route directly connecting the two. According to HOPI, however, the person would not be able to draw a map of the route, but would be able to point in the general direction of the target building and provide an approximate distance to reach it.

## Discussion

The limitations attributed to conditioning when it comes to explaining apparently complex spatial behaviors is often due to a simplistic conception of associative learning processes that are reduced to S-R learning and/or first-order CS-US associations. We demonstrated how higher-order conditioning enables flexible goal-directed behavior, even in a one-trial test consisting of a new situation never encountered during the learning phase. Higher-order conditioning (Pavlov, 1927) results in the association between a CS2 and a US that have never been physically paired and can thus lead to new adaptive behaviors in the absence of directly experiencing a CS2 and the US together. Associative learning theories involve the acquisition and retrieval of CS-US associations. Higher-order conditioning extends this process to associations between neutral stimuli or routes, thus enabling larger connected networks of associations. These interconnected associative networks in turn support inferences of novel relations between any two points or bits of information within the network, thereby enabling rapid and flexible navigation even through unfamiliar territory. Spatial integration and novel route taking are no longer the sole purview of cognitive map theories (i.e., the locale system), but now can be accounted for by bottom-up associative processes (i.e., the taxon system). Specifically, we showed how a combination of higher-order conditioning and path integration processes can result in the formation of a novel goal-directed vector without requiring a representation of this route. Furthermore, a place-cell based cognitive mapping strategy may fail on its own to generate novel routes (Duvelle et al., 2021). O’Keefe and Nadel (1978) proposed that the taxon system is involved in non-flexible navigation, such as response learning, while the locale system supports flexible behaviors such as taking a detour, a shortcut, or a novel route. We propose that the taxon/locale systems dichotomy is not realistic and should be abandoned, and that an allocentric map-like representation of the environment as formulated by cognitive map theories is not necessary nor sufficient for flexible navigation that involves taking novel routes. Place coding, however, does retain an important function in discriminating parts of familiar environments. Our analysis reveals that associative learning and path integration processes can play a much larger role in flexible navigation. The next challenge consists of experimentally addressing the hypotheses proposed in the present article, for example by adapting strategies where the use of goal-directed vectors would be neutralized (for a review, see Wehner, 2020) in tasks testing spatial integration and novel route taking, and to determine the underlying neural processes that support the processes elucidated by HOPI.

## Author Contributions

All authors listed have made a substantial, direct and intellectual contribution to the work, and approved it for publication.

## Conflict of Interest

The authors declare that the research was conducted in the absence of any commercial or financial relationships that could be construed as a potential conflict of interest.

## Publisher’s Note

All claims expressed in this article are solely those of the authors and do not necessarily represent those of their affiliated organizations, or those of the publisher, the editors and the reviewers. Any product that may be evaluated in this article, or claim that may be made by its manufacturer, is not guaranteed or endorsed by the publisher.

## Footnotes

1 We should also consider the possibility of path integration being performed virtually via a sequential activation of place cells covering the internal paths connecting D to C, resulting in a bidirectional vector that informs the direction and distance between D and C. While purely hypothetical, this explanation violates one of the criteria according to which cognitive mapping should not be explained by path integration.
